# Supplementary material for: Low illumination fog noise image denoising method based on ACE-GPM
Source: PLoS One. 2024 May 7;19(5):e0302492. doi: 10.1371/journal.pone.0302492 (PMC11075849; doi:10.1371/journal.pone.0302492)
Supplement: Attachment — Submitted filename: Response to Reviewers.pdf [file pone.0302492.s001.pdf]

Xingjian Wang
College of Computer and Control Engineering
Northeast Forestry University
Harbin, Heilongjiang, China
jianxingwang@126.com

December 1, 2023

Dear Editor/Reviewers,

I would like to express my gratitude for your thorough review of our manuscript, titled "Improved Image Enhancement by Partial Differential Equation and Automatic Color Equalization," submitted under the code PONE-D-23-30473. Your constructive feedback is invaluable, and we appreciate the opportunity to address the reviewers' comments and improve our work.

In our previous communication, we inadvertently provided an incorrect amendment to the financial disclosure statement. The accurate and updated statement is as follows: "This work was supported in part by Natural Science Foundation of Heilongjiang Province under Grant LH2020C048, and in part by the Harbin Science and Technology Innovation Talent Research Foundation under Grant 2017RAQXJ108." Funders played a vital role in supporting this research. Both funders were the main responsible persons for the project, contributing 10,0000 RMB each.

In response to the review, we have carefully considered each point raised and have outlined our revision plan below:

Reviewer 1:

I. Abstract:

1. The updated abstract reflects this paper's image reconstruction method steps. Firstly, an automatic color equalization algorithm(ACE) is used to improve the contrast of the low-light image containing noise. Secondly, the maximum between-cluster variance method(OTSU) was used for threshold segmentation. Then, according to the segmentation threshold, the image was enhanced by gradient using the enhancement function. Finally, the P-M model was used to denoise and enhance the gradient image.

2. The method proposed in this paper is mainly elaborated at the beginning of the third sentence of the abstract.

3. The title is updated to:Low illumination fog noise image denoising method based on ACE-GPM.

4. The comparison results between the proposed method and other methods are reflected in the abstract. Compared with other denoising methods, the ACE-GPM method proposed in this paper improves image contrast and removes noise while effectively retaining image details and texture information. The information entropy value is increased by 0.42 on average. In addition, the proposed method requires fewer computational resources while maintaining image quality.

II. Introduction:

In the introduction of the revised version, this paper first expounds on the common types of noise in digital images and the reasons for their generation. Subsequently, we explain the denoising methods proposed in the literature and their advantages and disadvantages. Finally, we show the proposed method's working block diagram and method steps so readers can better understand our method.

III. Method:

1. In the ninth paragraph of the introduction, this paper elaborates on the anisotropic P-M diffusion equation model and its advantages and disadvantages in image denoising and enhancement. This model has a wide range of application values in image processing, but due to its limitations, some areas also need improvement.

2. In the research method section of this paper, part D elaborates in-depth, and how to apply the OTSU method for gradient enhancement is introduced in detail. At the same time, we also clarify how the method ADAPTS to the P-M model and its embodiment of the performance improvement of the P-M model.

3. At the end of the introduction, this paper shows a simple working block diagram of the proposed method and elaborates each step in detail.

IV. Experiment:

1. In the revised manuscript's experimental part, this paper adds two sets of visual contrast and FOM merit evaluation to verify the effectiveness and reliability of the proposed method comprehensively. These additional experimental groups are intended to provide more exhaustive empirical support for our approach.

2. The revised manuscript details the hardware and software specifications in Section A of the experiment. The introduction section clearly describes the working steps of the proposed method. Each part of the research method constitutes the processing flow of the method in turn.

3. This paper studies the noise reduction problem of low-light fog images. We propose an effective denoising method for fog noise in low-light fog images.

4. This paper selects low-light fog noise images taken under natural conditions, and the comparison results between visual benchmark images and images processed by the method are provided in Section D of the experiment.

5. In Table 2 of the original paper, we detail the objective index evaluation of the images processed by various methods, including indicators such as average brightness, standard deviation, information entropy, peak signal-to-noise ratio (PSNR), and similarity index (SSIM). Because these evaluation metrics are not explained in detail in the original paper, it may have brought you questions. Therefore, in Section B of the experimental section of the revised manuscript, we present the FOM merit evaluation index and its calculation formula in this paper. In Tables 3, 4, and 5 in Section D, we detail the FOM merit evaluation index data of the processed images by each denoising algorithm.

6. According to the data in Table 2 of the original paper, the running time of vari-

ous methods for processing waterfall images may need to be more intuitive to show their effects. Therefore, after expert advice, this paper shows the line chart of running time for images with different resolutions in FIG. 7, part E of the experiment of the revised manuscript. This gives the reader a clearer picture of how different processing methods differ in running time and helps the reader evaluate the performance of each method more accurately.

7. The revised version of experiment section E shows the evaluation data of each part of the proposed method and the total time complexity.

8. In the previous version, the introduction of OTSU's algorithm may have some things that could be improved. Based on the expert feedback, this paper gives a more comprehensive description of the OTSU threshold method. It explains in detail how it is applied to the P-M model in Section B of the revised version of research methods.

9. With revisions and updates, the references and conclusions in this paper have been refined. In the references, we included some crucial articles published recently to ensure the timeliness and relevance of this paper. At the same time, in the conclusion section, we point out some limitations of the proposed method. We still need to adaptively select the P-M model's critical parameters for different pixel and definition images.

Reviewer 2:

A: In the revised version of Experiment Section D, we show the contrast effect clearly in the form of a table. See Table 2 for details.

B: In the experiment D section of the revised draft, we conduct multiple comparison experiments of eight existing denoising algorithms. We use the visual effect and FOM figure of merit as evaluation indicators to compare and evaluate the performance of various methods.

C: The format of the references has been corrected, and references have been made to recent journal articles to ensure the timeliness and relevance of this article.

D: For readers' convenience, this paper's work block diagram is provided in the revised draft, and the specific operation steps of each part are described in detail. Such an arrangement provides a more transparent and intuitive understanding, which helps readers better grasp the main content and ideas of this paper.

E: The proposed method is currently suitable for low-light fog images, so images with different sharpness (low contrast, high contrast, brightness) have yet to be applied to the algorithm for performance evaluation.

Review 3:

1. Abstract: We have revised and updated the paper's abstract according to the expert advice. Firstly, this paper leads to the proposed method for the problems of the existing P-M model. Secondly, the implementation of the method proposed in this paper is briefly described. Finally, the experimental results comparing the

proposed method with other denoising methods in the literature are described to prove the effectiveness of the proposed method.

2. Introduction: In the revised manuscript's introduction section, we describe this paper's workflow in detail and clearly explain the tasks of each stage. In this way, we can more accurately communicate our work's content, goals, and importance so that readers can better understand our work.

3. References Cited: After revision, all references cited in this paper have been updated to ensure timeliness and relevance.

4. Format and formula: After carefully reviewing and revising the first draft and referring to the reference articles provided by experts, we have revised the format and formula to ensure that the final revised draft meets the rigorous, stable, rational, and official requirements in form and content.

5. Flowchart of the paper framework: At the end of the introduction section of the revised manuscript, we attach a flowchart of the work of this paper, which details the tasks and objectives of each stage.

6. Image clarity: In the revised version, to more fully demonstrate the effectiveness of the proposed method, we conduct more comparative experiments and improve the image's contrast.

7. Ablation Experiment: In the revised version, the ablation experiment is added to Part F of an experiment to evaluate the influence of each part of the method and the combination of each part on the P-M model to reflect the effectiveness of the improved strategy in this paper.

Thank you again for your time and consideration. We look forward to the opportunity for our improved manuscript to be reconsidered for publication in PLOS ONE.

Kind regards,

Xingjian Wang
College of Computer and Control Engineering
Northeast Forestry University
