# Supplementary material for: Low illumination fog noise image denoising method based on ACE-GPM
Source: PLoS One. 2024 May 7;19(5):e0302492. doi: 10.1371/journal.pone.0302492 (PMC11075849; doi:10.1371/journal.pone.0302492)
Supplement: Attachment — Submitted filename: Response to Reviewers.pdf [file pone.0302492.s002.pdf]

Thank you for taking time out of your busy schedule to read my manuscript and for your reply letter. For the questions and opinions of the experts, I will reply one by one.

1. **Title** : This manuscript has been carefully revised, and the title has been precisely adjusted in response to the problem raised by the reviewers. The revised title is "Low illumination fog noise image denoising method based on ACE-GPM" to more accurately reflect this paper's research content and focus.

2. **Abstract**： Based on the reviewers' valuable comments, the “abstract ” part has been carefully optimized. In the abstract, we first point out the existing problems, clarify the main research methods adopted, and give a brief overview of the experimental results as the concluding part of the abstract. Such modifications help readers quickly understand this paper's core content and research results.

3. **Introduction**： After careful consideration of the reviewers' valuable comments on the introduction section, this manuscript has revised the introduction section accordingly. The revised content highlights the innovation of this paper even more and clarifies the overall architecture of the research methodology. We adopted the reviewers' suggestions to make this paper more rigorous, sedate, rational, and in line with the requirements of the official language style.

4. **Formula**： After careful consideration of the valuable suggestions of the reviewers on the formula, this revised version of the manuscript comprehensively reviewed and revised the formula involved and elaborated and explained the various parameters in the formula to ensure the logic and rigor of the paper.

5. **Framework flow chart**: After the reviewer pointed out the lack of a paper framework flow chart in the original manuscript, the revised version of this manuscript actively adopted the reviewer's suggestions. It referred to the literature resources recommended by the reviewer. After careful modification and improvement, we draw a detailed framework flow chart of the research method in this paper and give a comprehensive and in-depth explanation of the various components within the framework. Through these improvements, we expect to significantly improve the readability and intelligibility of the paper and provide readers with a more transparent and intuitive display of research ideas and methodologies. Refer to the last section of the introduction in the paper for a detailed framework flowchart and illustration.

6. **Low resolution**: After careful consideration and review, the revised version of this manuscript has been thoroughly checked and addressed, given the low resolution of the images raised by the reviewers. We have made the necessary optimizations and upgrades to the pictures to ensure their clarity and resolution reach a higher standard, thus more accurately conveying the research content and results. These improvements will contribute to the overall quality and readability of the manuscript.

7. **Ablation experiments**: After careful consideration of the valuable comments of the reviewers, we have supplemented the revised manuscript with ablation experiments. Specifically, we conduct relevant Settings in Section 3.5 of the paper and verify the effectiveness of the proposed improved scheme through an exhaustive analysis. This addition is intended to fully demonstrate the depth and breadth of the research while also providing readers with clearer and more robust evidence support.

8. **Spelling and grammatical**: In response to the reviewers' comments on spelling and grammatical errors in this manuscript, we will review the paragraphs one by one and accurately revise the sentences with grammatical errors to ensure the accuracy and fluency of the text.

9. **Conclusions**: After carefully considering the reviewers' comments on this paper's conclusions, we have decided to revise and improve the findings in the manuscript. The revised conclusion not only elaborates on the workflow and performance of the proposed method but also deeply analyzes the limitations of the proposed method and important directions for future research. Such revisions aim to improve the scientific and rigorous nature of the study and provide more accurate and comprehensive references for researchers in related fields.

10. **charts and graphs**: After careful study of the reviewers' feedback on the charts and graphs of the manuscript, we have decided to focus on the presentation of charts and tables in this revision and to improve their readability and accuracy through appropriate magnification and optimization measures.

11. **Reference**: Given the problem of inconsistent reference format raised by the reviewers, we have carried out a comprehensive revision of the reference section in the original manuscript. This revision aims to ensure that all cited documents follow a uniform formatting standard, thus improving the academic normativity and readability of the paper. We thank the reviewers for their valuable comments. We will continue to work hard to improve the quality of the paper.

12. **Introduction**: Based on the suggestions the reviewers put forward, this revised manuscript's introduction first summarizes the common noise types and their corresponding denoising algorithms. It makes it clear that the main research object of this paper is the low-light fog noise image. Through carefully designed experiments, we verify that the proposed method can effectively preserve the detailed texture information of the image while removing the fog noise in the picture, ensuring that the image quality is comprehensively improved.

13. **Time complexity**: Given the problem raised by the reviewers that the time complexity of the method needed to be evaluated in the manuscript, this revised manuscript has added a detailed analysis of the time complexity in Section 3.4.

14. **OTSU's threshold**: In response to the reviewer's feedback on the need for in-depth elaboration of OTSU's threshold, this revised manuscript elaborates on the specific steps of OTSU's algorithm in Section 2.2. It explores in depth the performance improvement that can be achieved by the algorithm in this study. We are committed to providing precise and detailed instructions to ensure readers fully understand the specific application and impact of OTSU's threshold in this study.

15. **FOM merit evaluation index**: In response to the reviewer's request for a more comprehensive evaluation of the method's performance in this paper, the revised manuscript has added multiple reliable performance evaluation indicators based on the original experimental setup. It includes peak mean square error (RMSE), Mean absolute error (AME), and mean square error of structural similarity (SDME). These new evaluation indexes will form a more complete evaluation system to reflect the method's performance in practical applications accurately.
